# Association of Heart-Type Fatty Acid-Binding Protein with Cardiovascular Risk Factors and All-Cause Mortality in the General Population: The Takahata Study

**DOI:** 10.1371/journal.pone.0094834

**Published:** 2014-05-21

**Authors:** Yoichiro Otaki, Tetsu Watanabe, Hiroki Takahashi, Atushi Hirayama, Taro Narumi, Shinpei Kadowaki, Yuki Honda, Takanori Arimoto, Tetsuro Shishido, Takuya Miyamoto, Tsuneo Konta, Yoko Shibata, Akira Fukao, Makoto Daimon, Yoshiyuki Ueno, Takeo Kato, Takamasa Kayama, Isao Kubota

**Affiliations:** 1 Department of Cardiology, Pulmonology, and Nephrology, Yamagata University School of Medicine, Yamagata, Japan; 2 Global Center of Excellence Program Study Group, Yamagata University School of Medicine, Yamagata, Japan; 3 Department of Endocrinology and Metabolism, Hirosaki University Graduate School of Medicine, Aomori, Japan; Washington Hospital Center, United States of America

## Abstract

**Background:**

Despite many recent advances in medicine, preventing the development of cardiovascular diseases remains a challenge. Heart-type fatty acid-binding protein (H-FABP) is a marker of ongoing myocardial damage and has been reported to be a useful indicator for future cardiovascular events. However, it remains to be determined whether H-FABP can predict all-cause and cardiovascular deaths in the general population.

**Methods and Results:**

This longitudinal cohort study included 3,503 subjects who participated in a community-based health checkup with a 7-year follow-up. Serum H-FABP was measured in registered subjects. The results demonstrated that higher H-FABP levels were associated with increasing numbers of cardiovascular risk factors, including hypertension, diabetes mellitus, obesity, and metabolic syndrome. There were 158 deaths during the follow-up period, including 50 cardiovascular deaths. Deceased subjects had higher H-FABP levels compared to surviving subjects. Multivariate Cox proportional hazard regression analysis revealed that H-FABP is an independent predictor of all-cause and cardiovascular deaths after adjustments for confounding factors. Subjects were divided into four quartiles according to H-FABP level, and Kaplan-Meier analysis demonstrated that the highest H-FABP quartile was associated with the greatest risks for all-cause and cardiovascular deaths. Net reclassification index and integrated discrimination index were significantly increased by addition of H-FABP to cardiovascular risk factors.

**Conclusions:**

H-FABP level was increased in association with greater numbers of cardiovascular risk factors and was an independent risk factor for all-cause and cardiovascular deaths. H-FABP could be a useful indicator for the early identification of high-risk subjects in the general population.

## Introduction

Despite technical advances in medicine, chronic heart failure remains a public health problem associated with high all-cause and cardiovascular mortality [1], [2]. According to the American College of Cardiology/American Heart Association (ACC/AHA) guideline, treatment of cardiovascular risk factors, such as hypertension, diabetes mellitus, obesity, and metabolic syndrome are recommended in subjects at high risk for developing stage A heart failure [3]. Therefore, early identification and risk-stratification of high-risk subjects in the general population would be helpful in preventing cardiovascular disease and subsequent premature deaths.

Cardiac biomarkers are generally used for the diagnosis or assessment of heart diseases [4], [5], [6], [7]. Recent studies demonstrated that cardiac biomarkers can predict an increased risk for death in subjects in the general population [8], [9]. Heart-type fatty acid-binding protein (H-FABP) is a low molecular weight protein in the cytosol of cardiomyocytes. H-FABP is rapidly released into the circulation from damaged myocardial tissue [10], which makes it a useful marker for ongoing myocardial damage. H-FABP levels can therefore be used to stratify risk for various heart diseases [11], [12], [13]. However, it remains to be determined whether serum H-FABP levels can predict cardiovascular diseases in the general population.

The purposes of the present study were to study the association of H-FABP levels with the presence of cardiovascular risk factors, and to determine whether H-FABP levels can predict all-cause and cardiovascular mortality in subjects from the general population.

## Methods

### Ethics Statement and Study population

The institutional ethics committee of Yamagata University School of Medicine approved the study, and all participants provided written informed consent. The procedures were performed in accordance with the Helsinki Declaration.

This study was a part of the ongoing Molecular Epidemiological Study, utilizing the resources of the Regional Characteristics of 21^st^ Century Centers of Excellence (COE) Program and the Global COE in Japan.

This study was based on a community-based annual health check-up of inhabitants from the town of Takahata in northern Japan (total population 26,026). Community members, aged >40 years were invited to participate. Between June 2004 and November 2007, 3,520 subjects (1,579 men and 1,941 women) were enrolled in the study. Subjects completed a self-reported questionnaire to document their medical history, current medication use, and clinical symptoms. Seventeen subjects were excluded due to incomplete data or study withdrawal.

### Measurement

Hypertension was defined as systolic blood pressure (BP) ≥140 mmHg, diastolic BP ≥90 mmHg, or antihypertensive medication use. Diabetes mellitus was defined as fasting blood glucose (FBG) ≥7.0 mmol/L, glycosylated hemoglobin A1c ≥6.5% (National Glyco hemoglobin Standardization Program), or anti-diabetic medication use. Hyperlipidemia was defined as total cholesterol ≥5.7 mmol/L, triglyceride ≥1.7 mmol/L, or anti-hyperlipidemic drug use. Obesity was defined as body mass index ≥25 kg/m^2^ [14]. Metabolic syndrome (Mets) was defined according to the modified National Cholesterol Education Program Adult Treatment Panel III (NCEP-ATP III) criteria, which require fulfilment of at least three of the five following: BMI ≥25 kg/m^2^, elevated triglyceride (TG) level (≥1.7 mmol/L), reduced level of high-density lipoprotein cholesterol (HDL-C; <1.03 mmol/L in men and <1.29 mmol/L in women), elevated FBG level (≥6.1 mmol/L) or previously diagnosed diabetes mellitus, and elevated BP (systolic BP ≥130 mmHg and diastolic BP ≥85 mmHg) or anti-hypertensive medication use [15], [16]. Chronic kidney disease (CKD) was defined as a reduced glomerular filtration rate (<60 mL/min/1.73m^2^) according to Kidney Disease Outcomes Quality Initiative clinical guideline [17], [18]. Electrocardiographic left ventricular hypertrophy was diagnosed by a cardiologist according to the Minnesota code (1982 revised edition).

### Biochemical markers

Blood samples for measurements of serum H-FABP concentrations were drawn and centrifuged at 2,500 *g* for 15 min at 4°C within 30 min of collection, and the obtained serum was stored at −70°C until analysis. H-FABP levels were measured using a two-step sandwich enzyme-linked immunosorbent assay (ELISA) kit (MARKIT-M H-FABP, Dainippon Pharmaceutical Co. Ltd., Tokyo, Japan), as previously described [19]. Detection limit, measurement range, and reference interval of H-FABP were 0–250 ng/mL, 1.0–37, <6.2 ng/mL, respectively.

Blood samples were also obtained for measuring brain natriuretic peptide (BNP). These samples were transferred to chilled tubes containing 4.5 mg ethylenediaminetetraacetic acid disodium salt and aprotinin (500 U/mL), and centrifuged at 1,000 *g* for 15 minutes at 4°C. The clarified plasma samples were frozen, stored at −70°C, and thawed just before the assay was performed. BNP concentrations were measured using a commercially available radioimmunoassay specific for human BNP (Shiono RIA BNP assay kit, Shionogi Co. Ltd., Tokyo, Japan) [20], [21]. Detection limit, measurement range, and reference interval of BNP were 0–2902, 3.4–1392, <18.4 pg/mL, respectively.

Estimated glomerular filtration rate (eGFR) was calculated by the modification of diet in renal disease (MDRD) equation with the Japanese coefficient [22].

Insulin resistance was defined as elevated homeostasis model assessment ratio (>2.5). Homeostasis model assessment ratio was calculated by the following equation: (FBG ×fasting insulin)/405 [23].

Glycosylated hemoglobin A1c, FBG, total cholesterol, TG, and HDL cholesterol, and creatinine were measured by standard method as illustrated in Table S1.

### Endpoint and follow-up

All subjects were prospectively followed for a median period of 2,124 days (interquartile range, 1,972–2,343 days). The endpoint was all-cause death, which was also broken down into cancer death, cardiovascular death, lung disease death including pneumonia, and others. Cardiovascular death was defined as death due to coronary artery disease, heart failure, arrhythmia, stroke, and aortic artery disease. The cause of death was determined by reviewing death certificates through the end of 2010. The death code (International Classification of Diseases, 10^th^ Revision) and the data place of death were reviewed.

### Statistical analysis

Normality of continuous variables was checked by a Kolmogorov-Smirnov-Lillefors test. Since H-FABP and BNP were not normally distributed, we used log_e_ H-FABP and log_e_ BNP for all analyses. All values are expressed as the mean ± standard deviation. Continuous and categorical variables were compared with t-tests and chi-square tests, respectively. Kruskal-Wallis test was used to compare cardiovascular risk number with H-FABP levels. A Cox proportional hazard analysis was performed to determine independent predictors for all-cause deaths, and cardiovascular risk factors and significant predictors selected in the univariate analysis were entered into the multivariate analysis. To determine the independent predictors for cardiovascular deaths, cardiovascular risk factors and significant predictors selected in the univariate analysis were entered into multivariate analysis in a stepwise manner. The receiver operating characteristics (ROC) curves for all-cause deaths were constructed and used as a measure of the predictive accuracy of H-FABP and BNP on all-cause deaths. In addition, we calculated the net reclassification index (NRI) and the integrated discrimination index (IDI) to measure the quality of improvement for the correct reclassification according to the addition of H-FABP to the model. The interaction between H-FABP and BNP levels was analyzed by Cox proportional hazard regression analysis, using H-FABP and BNP cut-off values. Differences among four groups based on H-FABP quartiles were analyzed by analysis of variance (ANOVA) with Scheffe's post hoc tests. Survival curves were constructed with the Kaplan-Meier method and compared using log-rank tests. A value of P<0.05 was considered statistically significant. All statistical analyses were performed with a standard program package (JMP version 8; SAS Institute Inc., Cary, NC, USA and R 3.0.2 with additional packages including Rcmdr, Epi, pROC, and PredictABEL).

## Results

### Comparison of clinical characteristics between surviving and deceased subjects

The subject's baseline characteristics are shown in Table 1. There were 1,570 men and 1,933 women. The mean log_e_ H-FABP value was 1.25 ng/mL. Subjects who died during the course of the study were older and had higher prevalence rates of previous cardiovascular diseases, smoking, hypertension, diabetes mellitus, CKD, and atrial fibrillation (AF) than those who survived. The deceased subjects had higher systolic BP, glycosylated hemoglobin A1c, FBG, BNP, and H-FABP levels than those who did not. eGFR was lower in deceased subjects than in those who survived.

**Table 1:** Clinical characteristics of subjects with and without all-cause deaths.

Variables	All subject n = 3503	All-cause deaths(−) n = 3345	All-cause deaths(+) n = 158	P value
Age, years	63±10	62±10	73±8	<0.0001
Men/women, n	1570/1933	1455/1890	115/43	<0.0001
Obesity, n (%)	1035 (30%)	996 (30%)	39 (25%)	0.1704
Previous CVD, n (%)	461 (13%)	427 (13%)	34 (22%)	0.0015
Previous cancer, n (%)	74 (2.1%)	70 (2.1%)	4 (2.5%)	0.7077
Smoking, n (%)	1126 (32%)	1052 (31%)	74 (47%)	<0.0001
Hypertension, n (%)	1285 (37%)	1202 (36%)	83 (53%)	<0.0001
Diabetes mellitus, n (%)	243 (10%)	223 (7%)	20 (13%)	0.0038
Hyperlipidemia, n (%)	1361 (39%)	1301 (39%)	60 (38%)	0.8168
CKD, n (%)	235 (7%)	210 (6%)	25 (16%)	<0.0001
MetS, n (%)	454 (13%)	431 (13%)	23 (15%)	0.5409
Insulin resistance	354 (11%)	333 (11%)	21 (15%)	0.1180
Electrocardiographic LVH, n (%)	143 (4.1%)	135 (4%)	8 (5.1%)	0.5236
AF, n (%)	52 (1.5%)	38 (1.1%)	14 (9%)	<0.0001
Systolic BP, mmHg	134±16	134±16	138±18	0.0019
Diastolic BP, mmHg	80±10	80±10	80±11	0.9799
HbA1c, %	5.7±0.7	5.7±0.7	5.8±0.8	0.0065
FBG, mmol/L	5.27±0.94	5.27±0.94	5.50±0.83	0.0010
eGFR, mL/min/1.73 m^2^	82±16	82±16	74±18	<0.0001
Log_e_ BNP, pg/mL	3.00±0.85	2.97±0.83	3.54±1.11	<0.0001
Log_e_ H-FABP, ng/mL	1.25±0.43	1.23±0.43	1.52±0.49	<0.0001

Data are expressed as mean ± standard deviation or number (%).

CVD, cardiovascular disease; CKD, chronic kidney disease; MetS, metabolic syndrome; LVH, left ventricular hypertrophy; AF, atrial fibrillation; BP, blood pressure; HbA1c, glycosylated hemoglobin A1c, FBG, fasting blood glucose; eGFR, estimated glomerular filtration rate; BNP, brain natriuretic peptide; H-FABP, heart type fatty acid binding protein.

### Association between H-FABP levels and cardiovascular risk factors for stage A heart failure

**Figure 1:**
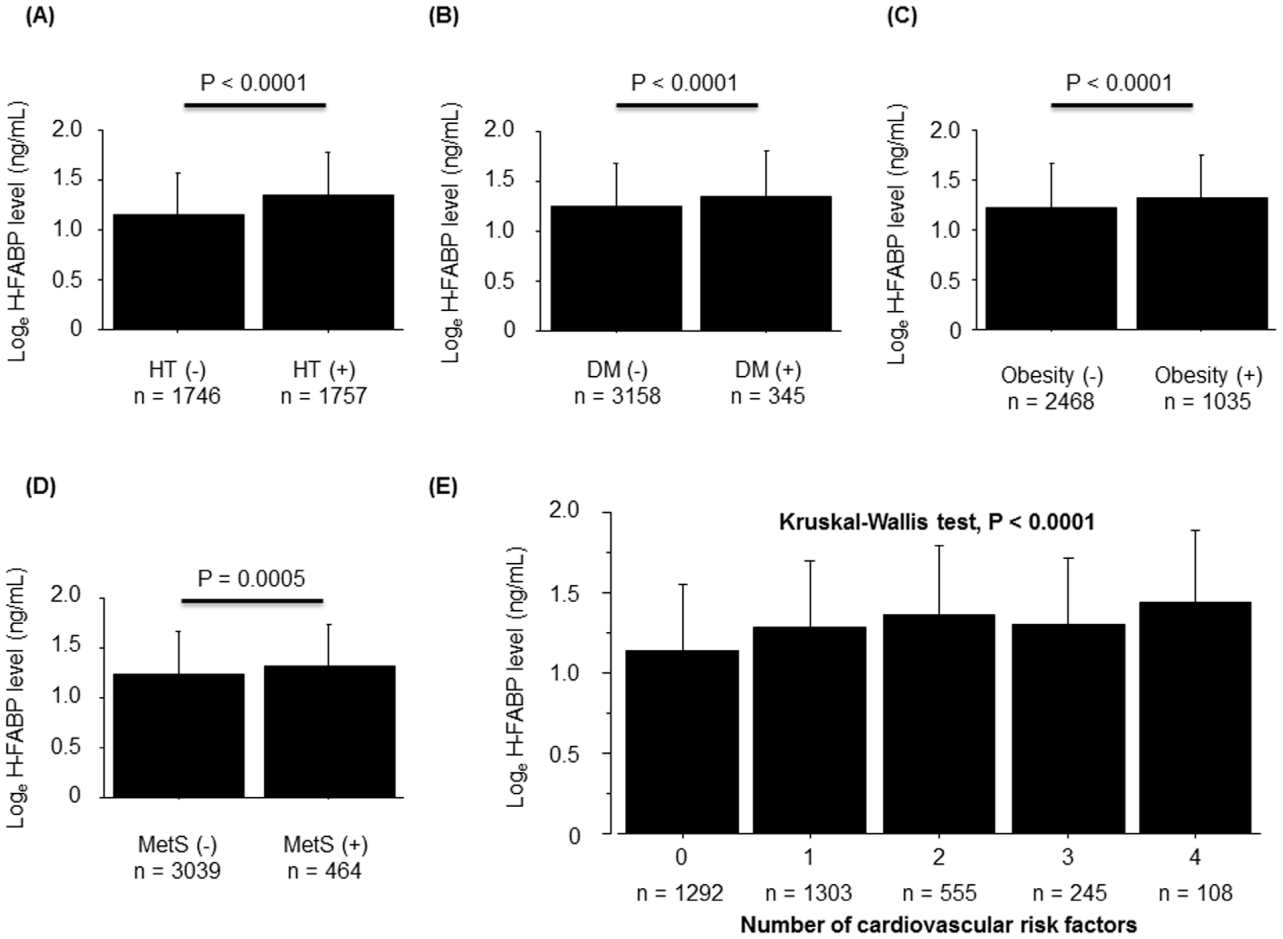
Associations between H-FABP levels and cardiovascular risk factors in the general population. H-FABP levels were higher in subjects with hypertension (**A**), diabetes mellitus (**B**), obesity (**C**), and metabolic syndrome (**D**). (**E**) Higher H-FABP levels were associated with greater numbers of cardiovascular risk factors (Kruskal-Wallis test, P<0.0001). HT, hypertension; DM, diabetes mellitus; MetS, metabolic syndrome.

As shown Figure 1, subjects who had each cardiovascular risk factor (hypertension, diabetes mellitus, obesity, and metabolic syndrome) showed higher H-FABP levels compared with those who did not. In addition, H-FABP levels increased with higher numbers of these cardiovascular risk factors, suggesting that subjects at higher risk for stage A heart failure had higher H-FABP levels than those with lower risk.

### All-cause and cardiovascular mortality and H-FABP

During the follow-up period, there were 158 all-cause deaths including 50 cardiovascular deaths, 74 cancer deaths, 20 lung disease deaths, and 14 other cause deaths.

**Figure 2:**
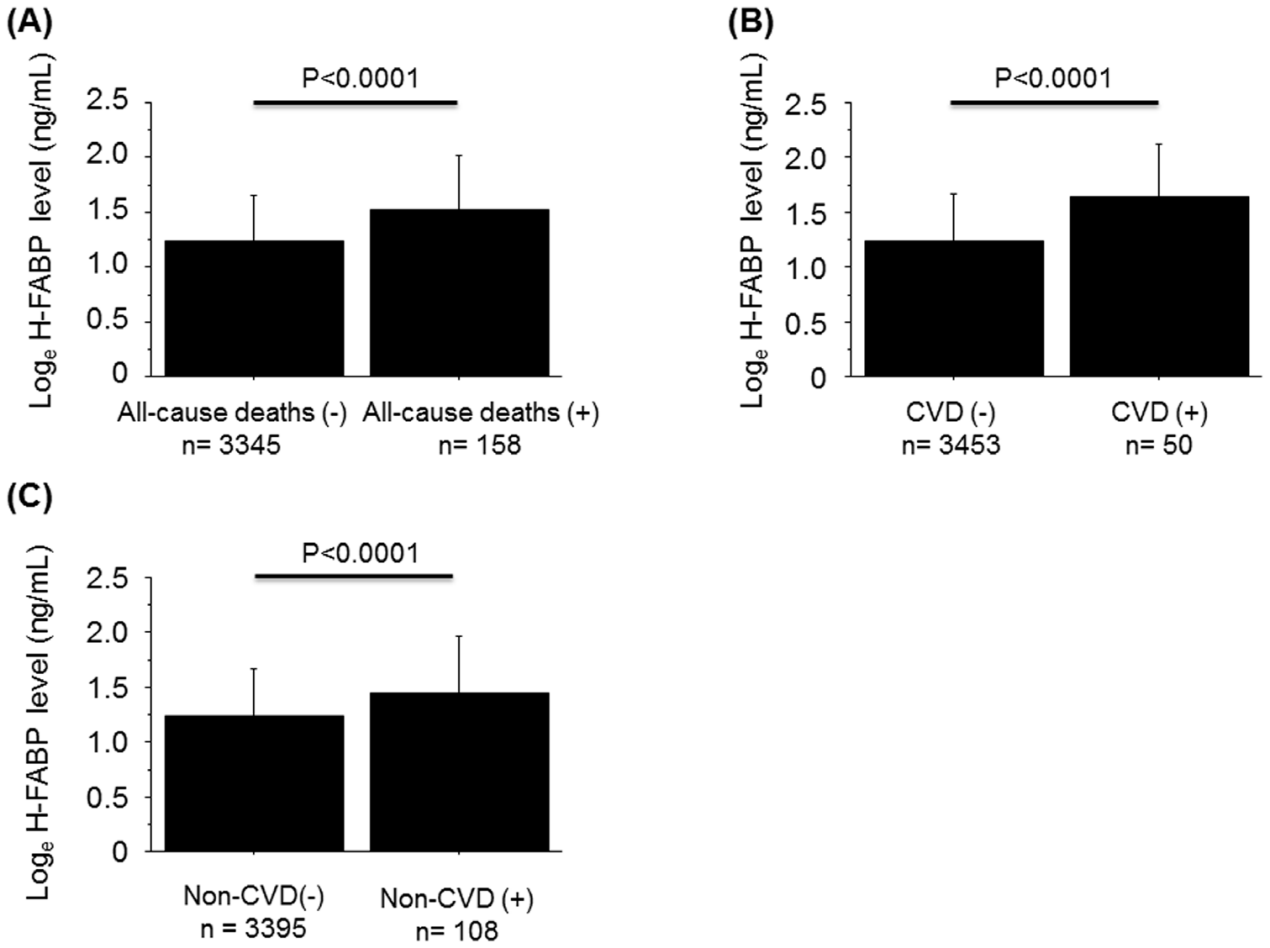
Associations between H-FABP levels and all-cause deaths, cardiovascular deaths, and non-cardiovascular deaths.

As shown in Figure 2, subjects who died during the course of the study had higher H-FABP levels compared to those who did not.

**Figure 3:**
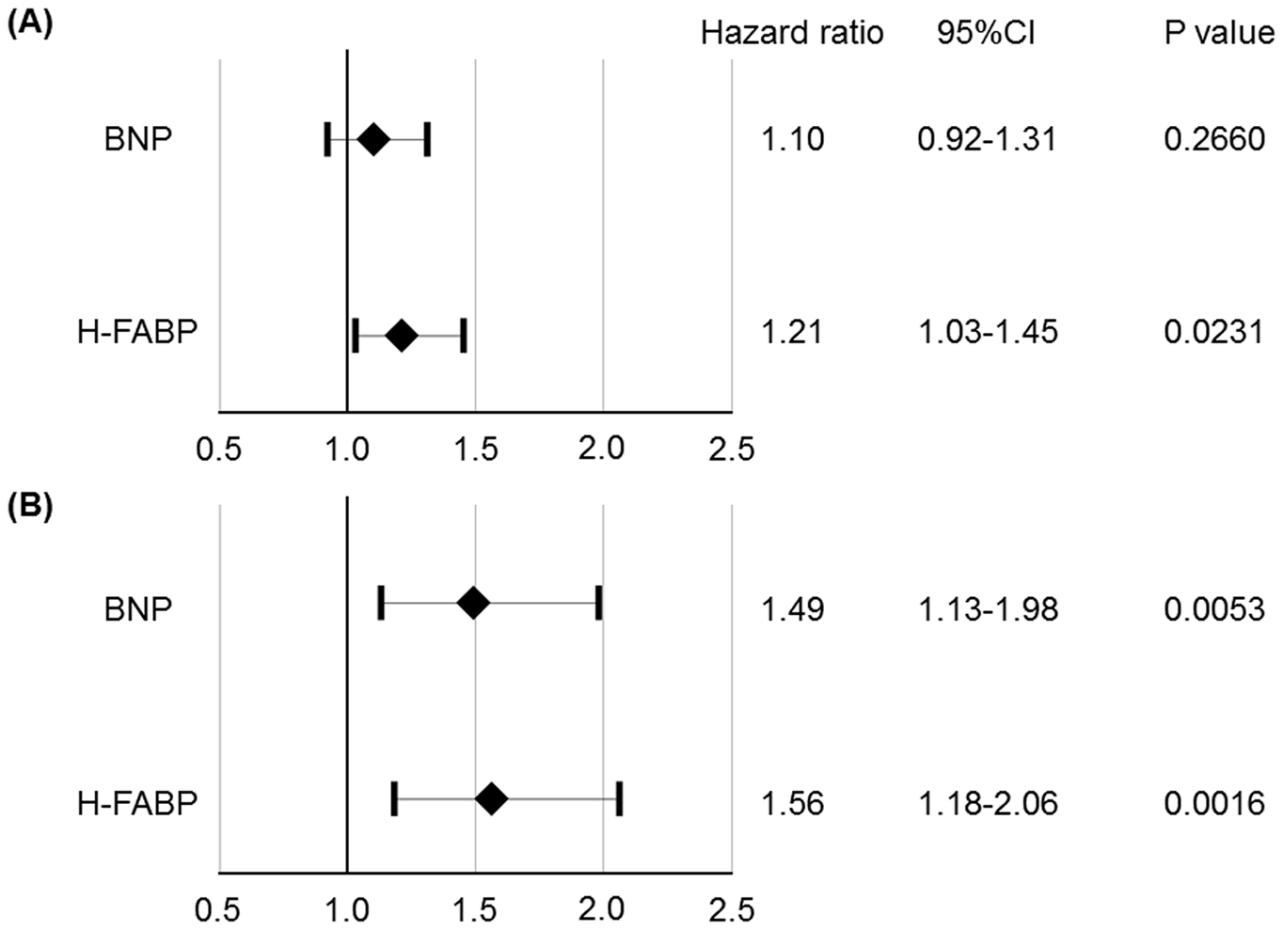
Multivariate Cox proportional hazard regression analysis for predicting all-cause deaths (A) and cardiovascular deaths (B). (**A**) Hazard ratios after adjustment for age, gender, smoking, previous cardiovascular disease, obesity, hypertension, diabetes mellitus, metabolic syndrome, chronic kidney disease, atrial fibrillation, and BNP. (**B**) Hazard ratio after adjustment for age, gender, atrial fibrillation and BNP.

To determine the risk factors for predicting all-cause deaths, we performed univariate and multivariate Cox proportional hazard regression analyses. In the univariate analysis, H-FABP was significantly associated with all-cause mortality (Table 2). Age, male gender, previous cardiovascular disease, smoking, hypertension, diabetes mellitus, CKD, AF, systolic BP, glycosylated hemoglobin A1c, FBG, eGFR, and BNP were also related to all-cause mortality. A multivariate Cox proportional hazard regression analysis demonstrated that H-FABP was an independent predictor of future all-cause mortality after adjusting for age, gender, smoking, previous cardiovascular disease, hypertension, diabetes mellitus, obesity, MetS, CKD, AF, and BNP (hazard ratio, 1.21; 95% confidence interval, 1.03–1.45; P = 0.0231; Figure 3A). A second, age, gender, smoking, previous cardiovascular disease, hypertension, diabetes mellitus, obesity, MetS, CKD, AF, BNP, and H-FABP were entered into the stepwise multivariate Cox proportional hazard regression analysis and it demonstrated that H-FABP was an independent predictor of future cardiovascular mortality (hazard ratio, 1.56; 95% confidence interval, 1.18–2.06; P = 0.0016; Figure 3B).

**Table 2:** Univariate Cox proportional hazard analysis for all-cause mortality.

Variables	Hazard ratio	95%CI	P Value
Age	1.13	1.10–1.15	<0.0001
Men/women	3.44	2.43–4.89	<0.0001
Obesity	1.31	0.92–1.89	0.1362
Previous CVD	1.74	1.19–2.54	0.0042
Smoking	1.94	1.42–2.64	<0.0001
Hypertension	1.82	1.33–2.49	0.0002
Diabetes mellitus	1.92	1.20–3.07	0.0063
Hyperlipidemia	1.00	0.73–1.39	0.9805
CKD	2.74	1.79–4.20	<0.0001
MetS	1.13	0.73–1.77	0.5734
Insulin resistance	1.35	0.85–2.15	0.2039
Electrocardiographic LVH	1.30	0.68–2.68	0.4753
AF	7.23	4.17–12.51	<0.0001
Systolic BP	1.02	1.01–1.03	0.0010
Diastolic BP	1.00	0.99–1.02	0.7909
HbA1c	1.28	1.10–1.48	0.0011
FBG	1.01	1.01–1.02	0.0002
eGFR(Per 1-SD increase)	0.59	0.49–0.70	<0.0001
BNP (Per 1-SD increase)	1.71	1.50–1.96	<0.0001
H-FABP (Per 1-SD increase)	1.72	1.51–1.96	<0.0001

CVD, cardiovascular disease; CKD, chronic kidney disease; MetS, metabolic syndrome; LVH, left ventricular hypertrophy; AF, atrial fibrillation; BP, blood pressure; HbA1c, glycosylated hemoglobin A1c; FBG, fasting blood glucose; eGFR, estimated glomerular filtration rate; BNP, brain natriuretic peptide; H-FABP, heart type fatty acid binding protein.

### Risk stratification

**Figure 4:**
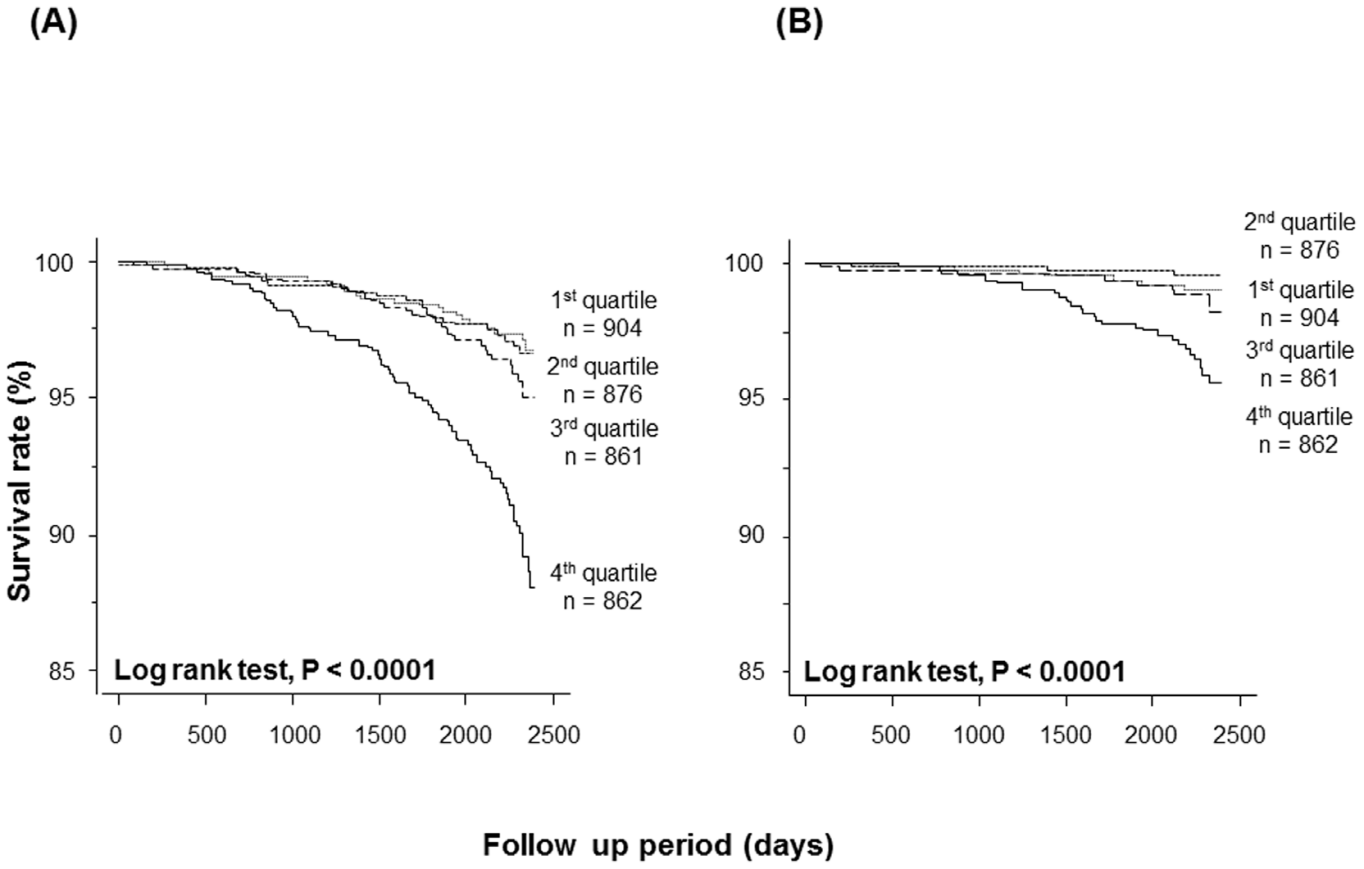
Kaplan-Meier analysis of all-cause deaths (A) and cardiovascular deaths (B) among subjects in all H-FABP quartiles.

All subjects were divided into quartiles according to H-FABP level: first quartile (≤0.96 ng/mL, n = 904), second quartile (0.96–1.22 ng/mL, n = 876), third quartile (1.22–1.50 ng/mL, n = 861), and fourth quartile (>1.50 ng/mL, n = 862). As shown in Table 3, subjects in the highest H-FABP quartile were older and had higher prevalence rates of obesity, previous cardiovascular disease, hypertension, diabetes mellitus, CKD, MetS, insulin resistance, electrocardiographic left ventricular hypertrophy, and AF, compared with the other three groups. Subjects in the highest quartile also showed lower eGFR and higher systolic BP and BNP compared with the other three groups. The mean glycosylated hemoglobin A1c was significantly greater in fourth quartile than in first and second quartiles. FBG was higher in the fourth quartile than in the first quartile. Subjects in the third quartile were older and showed higher systolic BP and lower eGFR than the first and second quartiles. Glycosylated hemoglobin A1c and FBG were higher in the third quartile than in the first quartile. Subjects in the second quartile were older and had higher systolic BP and lower eGFR than the first quartile. Kaplan-Meier analysis demonstrated that both all-cause and cardiovascular mortalities were the highest in fourth quartile compared with other three groups (Figure 4).

**Table 3:** Clinical characteristics among subjects with 1^st^ to 4^th^ quartile.

Variables	1^st^ quartile n = 904	2^nd^ quartile n = 876	3^rd^ quartile n = 861	4^th^ quartile n = 862
Age, years	57±9	61±10*	65±9*^†^	69±9*^†‡^
Men/women, n	348/556	373/503	422/439	427/435^§^
Obesity, n (%)	203 (22%)	247 (28%)	272 (32%)	313 (36%)^§^
Previous CVD, n (%)	71 (8%)	94 (11%)	135 (15%)	161 (19%)^§^
Previous cancer, n (%)	23 (2.5%)	15 (1.7%)	16 (1.9%)	20 (2.3)
Smoking, n (%)	293 (32%)	281 (32%)	271 (32%)	281 (33%)
Hypertension, n (%)	207 (23%)	278 (32%)	356 (41%)	444 (52%)^§^
Diabetes mellitus, n (%)	54 (6%)	53 (6%)	55 (6%)	81 (9%)^§^
Hyperlipidemia, n (%)	371 (41%)	369 (42%)	298 (35%)	323 (37%)^§^
CKD, n (%)	9 (1%)	26 (3%)	47 (6%)	153 (18%)^§^
MetS, n (%)	87 (10%)	120 (14%)	103 (12%)	144 (32%)^§^
Insulin resistance	59 (7%)	97 (12%)	93 (12%)	105 (13%)^§^
Electrocardiographic LVH, n (%)	35 (4%)	27 (3%)	31 (4%)	50 (6%)^§^
AF, n (%)	3 (0.3%)	12 (1.4%)	17 (2.0%)	20 (2.3%)^§^
Systolic BP, mmHg	130±16	133±15*	136±15*^†^	138±16*^†‡^
Diastolic BP, mmHg	79±10	80±10	80±10	80±10
HbA1c, %	5.6±0.7	5.7±0.7	5.7±0.7*	5.8±0.7*^†^
FBG, mmol/L	5.16±0.78	5.22±0.99	5.33±0.99*	5.38±0.94*
eGFR, mL/min/1.73 m^2^	90±16	83±15*	79±14*^†^	73±16*^†‡^
Log_e_ BNP, pg/mL	2.78±0.71	2.91±0.81*	3.05±0.89*^†^	3.26±0.93*^†‡^

Data are expressed as mean ± standard deviation or number (%).

CVD, cardiovascular disease; CKD, chronic kidney disease; Mets, metabolic syndrome; LVH, left ventricular hypertrophy; AF, atrial fibrillation; BP, blood pressure; HbA1c, glycosylated hemoglobin A1c, FBG, fasting blood glucose; eGFR, estimated glomerular filtration rate; BNP, brain natriuretic peptide; H-FABP, heart type fatty acid binding protein. *p<0.05 vs. 1^st^ quartile, ^†^p<0.05 vs. 2^nd^ quartile, ^‡^p<0.05 vs. 3^rd^ quartile by analysis of variance (ANOVA) with Scheffe post hoc test. ^§^p<0.05 by chi-square test.

### Combination of H-FABP and BNP

**Figure 5:**
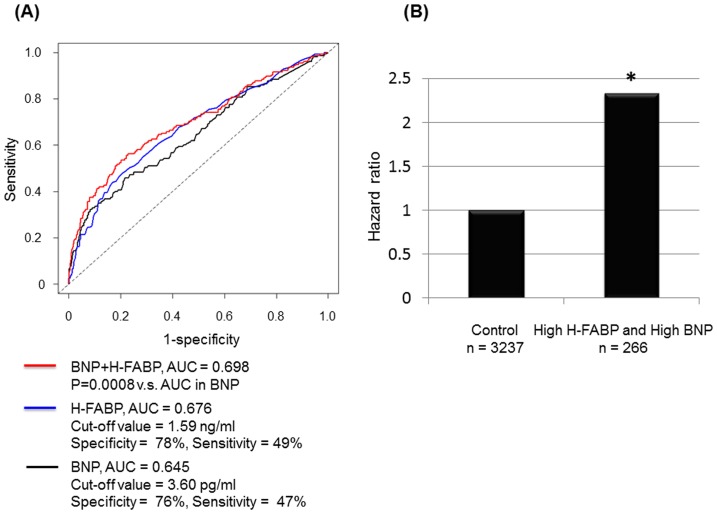
Combination of H-FABP and BNP. (**A**) The ROC curves of H-FABP, BNP, and H-FABP+BNP for all-cause deaths. (**B**) The statistical interaction between H-FABP and BNP. Subjects with high log_e_ H-FABP (≥1.59 ng/mL) and high log_e_ BNP (≥3.6 pg/mL) were at increased risk for all-cause death after adjustments for high H-FABP and high BNP (hazard ratio, 2.39; 95% confidence interval, 1.19–4.37; P = 0.0126).

To examine whether H-FABP improves the prognostic capacity of BNP, ROC analysis were performed. AUC of H-FABP, sensitivity, and specificity were 0.676, 78%, and 49%, respectively. The cut-off value of log_e_ H-FABP was 1.59 ng/mL. AUC of BNP, sensitivity, and specificity were 0.645, 76%, and 47%, respectively. The cut-off value of log_e_ BNP was 3.6 pg/mL. There was no significant difference in AUC of H-FABP and that of BNP. However, the AUC of BNP was significantly improved by addition of H-FABP (Figure 5A). Also, NRI and IDI were significantly improved in model with H-FABP than those with BNP only (NRI, 0.4498; 95% confidence interval, 0.2925–0.6071; P<0.0001 and IDI, 0.0209; 95% confidence interval, 0.0129–0.0292; P<0.0001). Next, we studied the statistical interaction between H-FABP and BNP. As shown in Figure 5B, multivariate Cox proportional hazard regression analysis demonstrated that subjects with high H-FABP (≥1.59 ng/mL) and high BNP (≥3.6 pg/mL) were at significantly increased risk for all-cause death after adjustments for high H-FABP and high BNP (hazard ratio, 2.39; 95% confidence interval, 1.19–4.37; P = 0.0126).

### Improvement of reclassification by addition of H-FABP to predict all-cause mortality

To examine whether model fit and discrimination improve with addition of H-FABP or BNP to the basic predictors such as age, HT, DM, obesity, and MetS, we evaluated the improvement of NRI and IDI. As shown in Table 4, NRI and IDI were significantly improved by addition of H-FABP. On the other hand, there was no significant difference in NRI by addition of BNP to the basic predictors.

**Table 4:** Statics for model fit and improvement with the addition of H-FABP on the prediction of all-cause mortality.

	AUC (P value)	NRI (95%CI, P value)	IDI (95%CI, P value)
Age+HT+DM+Obesity+Mets	0.793	Reference	Reference
Age+HT+DM+Obesity+Mets+BNP	0.793 (P = 0.9980)	0.0556 (−0.1053–0.2165, P = 0.4982)	0.0057 (0.0014–0.0100, P = 0.0101)
Age+HT+DM+Obesity+Mets+H-FABP	0.798 (P = 0.3374)	0.1632 (0.0023–0.324, P = 0.0467)	0.0073 (0.0022–0.0125, P = 0.0051)

AUC, area under the curve; 95%CI, 95% confidence interval; NRI, net reclassification index; IDI, integrated discrimination index; HT, hypertension; DM, diabetes mellitus; Mets, metabolic syndrome; BNP, brain natriuretic peptide; H-FABP, heart type fatty acid binding protein.

## Discussion

### Main findings

The results of this study revealed four novel findings: 1) H-FABP was detectable in all subjects and was higher in deceased subjects than in surviving subjects; 2) increased H-FABP values were associated with greater numbers of cardiovascular risk factors; 3) multivariate Cox proportional hazard regression analysis revealed that high H-FABP levels independently predicted future all-cause and cardiovascular mortality; and 4) Kaplan-Meier analysis demonstrated that all-cause and cardiovascular mortality were greatest in subjects in the highest H-FABP quartile.

### Latent myocardial damage assessed by H-FABP

Research into cardiac biomarkers has shifted toward identifying subjects in the general population who are at high risk. Previous studies have reported that BNP, troponin I, and troponin T are useful indicators of all-cause deaths and cardiovascular deaths in the general population [5], [8], [24], [25]. Although H-FABP is also a diagnostic marker of myocardial infarction [26], there has been no clinical research to reveal the prognostic value of H-FABP in general population.

Myocardial damage plays a key role in the progression of left ventricular remodeling in heart failure [27],[28]. Troponin leakage detected by a highly sensitive assay kit was reported to occur in 20–25% of the general population and 60% of elderly adults [24], [25], [29]. Importantly, H-FABP was detectable in all subjects who participated in this study. Studies in rats and clinical research with human autopsy cases have shown that H-FABP leakage occurs in the absence of myocyte necrosis [30]. Because H-FABP is a low molecular weight protein, cytosolic H-FABP is easily released into the circulation through the porous membranes of damaged myocardial cells [26]. Therefore, H-FABP is a sensitive marker for detecting latent myocardial damage.

### Associations of H-FABP with cardiovascular risk factors

Myocardial damage in an apparently healthy population may result from several chronic disease states such as CKD, subclinical myocardial infarction, coronary artery disease, and heart failure [29], [31]. In the present study, myocardial damage assessed by H-FABP was observed in the presence of cardiovascular risk factors, such as hypertension, diabetes mellitus, obesity and metabolic syndrome. The underlying mechanism of latent myocardial damage was reported to be involved in renin-angiotensin-aldosterone system activation, sympathetic nervous activation, and insulin resistance [32], [33], [34], [35], [36]. Interestingly, latent myocardial damage was more severe in subjects with higher numbers of cardiovascular risk factors in the present study. These findings suggest that H-FABP is a useful marker for detecting subjects at high risk for developing structural heart diseases (stage B heart failure).

### Clinical outcomes and H-FABP in the general population

Increased concentration of cardiac biomarker has been shown to associate with an increased risk for cardiovascular disease and subsequent high mortality in general population [8], [24], [37]. Similarly, our results show for the first time that H-FABP is a feasible marker for all-cause and cardiovascular deaths in the general population. Given that heart failure caused by HT, DM, and ischemic heart disease was significantly increased in Asian countries [38], it was plausible that H-FABP predicted future cardiovascular deaths in general population. Since this was the prospective cohort study, we did not confirm the precise mechanism by which circulating H-FABP level was related to all-cause mortality including cancer deaths. Subjects with elevated H-FABP had higher prevalence of HT, DM, obesity, Mets, and CKD, which are risks for all-cause deaths and cancer deaths [39], [40], [41]. Renin-angiotensin-aldosterone system activation and insulin resistance were also risks for the development of cancer in addition to worsening myocardial damage [42], [43]. These findings contributed to the fact that elevated H-FABP levels were significantly associated with future all-cause mortality as well as cardiovascular deaths.

H-FABP is reportedly a useful indicator of future cardiac prognosis independent of BNP in patients with CHF [20], [44]. Similarly, we found a statistically significant interaction between H-FABP and BNP that predicted future all-cause deaths, indicating that the combination of H-FABP and BNP evaluations would be useful in determining risk in the general population. Although 92% of subjects were within the normal range, H-FABP can be used to risk-stratify subjects in the general population based on their risk for all-cause and cardiovascular death.

### Limitation

This study was conducted with a large number of participants and had a long follow-up period, suggesting that results are reliable. However, there are some limitations. First, this study collected baseline information at a single time point. Subsequent medical interventions may have affected serum H-FABP levels. Second, non-fatal diseases were not assessed, which could result in an underestimation of the association between H-FABP levels and clinical outcomes. Third, because structural heart disease was not diagnosed by echocardiogram, we could not confirm the association between H-FABP level and heart disease. Fourth, since H-FABP level is elevated due to disordered elimination of H-FABP in subjects with kidney dysfunction, we could not completely eliminate effect of kidney dysfunction on H-FABP level. However, 93% of subjects had normal eGFR in the present study. Finally, the low rate of cardiovascular mortality observed in this study may primarily result from low prevalence of ischemic heart disease in Japan. The prevalence of ischemic heart disease is reportedly markedly lower in Japan compared with the western countries [45]. The rate of cardiovascular mortality in this study is thought to be equivalent to that seen in Japanese population based registry [46].

## Conclusions

Serum H-FABP is increased in subjects with increased numbers of cardiovascular risk factors. Notably, H-FABP could predict all-cause and cardiovascular mortality in subjects in the general population, suggesting that it is a promising marker to risk-stratify apparently healthy general populations.

## Supporting Information

Table S1 **Measurement method for HbA1c, Creatinine, total cholesterol, triglyceride, and HDL cholesterol.**
(DOCX)
