# Supplementary material for: Association of Heart-Type Fatty Acid-Binding Protein with Cardiovascular Risk Factors and All-Cause Mortality in the General Population: The Takahata Study
Source: PLoS One. 2014 May 21;9(5):e94834. doi: 10.1371/journal.pone.0094834 (PMC4029574; doi:10.1371/journal.pone.0094834)
Supplement: Table S1 — Measurement method for HbA1c, Creatinine, total cholesterol, triglyceride, and HDL cholesterol. (DOCX) [file pone.0094834.s001.docx]

**Supplemental table.** Measurement method for HbA1c, Creatinine, total cholesterol, triglyceride, and HDL cholesterol.

|  | Method | Reference interval |
| --- | --- | --- |
| HbA1c | HPLC method | 4.5-6.2 % |
| FBG | Enzyme electrode method | 3.9-6.1, mmol/L |
| Total cholesterol | Cholesterol oxidase enzyme method | 3.3-5.7, mmol/L |
| Triglyceride | Enzyme method | 0.3-1.7, mmol/L |
| HDL cholesterol | Chemically modified enzyme method | 1.1-2.5, mmol/L |
| Creatinine | Enzyme method | 54-92, μmol/L in man,  42-70, μmol/L in women |

HbA1c, glycosylated hemoglobin A1c; FBG, fasting blood glucose; HDL cholesterol, high density lipoprotein cholesterol.
